# The Comparative Efficacy of Non-ergot Dopamine Agonist and Potential Risk Factors for Motor Complications and Side Effects From NEDA Use in Early Parkinson's Disease: Evidence From Clinical Trials

**DOI:** 10.3389/fnagi.2022.831884

**Published:** 2022-04-22

**Authors:** Chunxiao Wu, Hongji Guo, Yingshan Xu, Luping Li, Xinyu Li, Chunzhi Tang, Dongfeng Chen, Meiling Zhu

**Affiliations:** ^1^Shenzhen Hospital of Integrated Traditional Chinese and Western Medicine, Guangzhou University of Chinese Medicine, Guangdong, China; ^2^The Research Center of Basic Integrative Medicine, Guangzhou University of Chinese Medicine, Guangzhou, China; ^3^Clinical Medical College of Acupuncture, Moxibustion and Rehabilitation, Guangzhou University of Chinese Medicine, Guangzhou, China

**Keywords:** non-ergot dopamine agonist, motor complications, Parkinson's disease, risk factors, dose response

## Abstract

**Background/Objectives:**

Non-ergot dopamine agonist (NEDA) are recommended as the first-line treatment for patients with early Parkinson's disease (PD) because of their efficacy in treating PD motor symptoms. However, systematic evaluations of the risk of motor complications induced by NEDA and risk factors potentially associated with motor complications are still lacking.

**Methods:**

Medline, Embase, the Cochrane Central Register of Controlled Trials, and Web of Science were searched for potentially eligible randomized controlled trials. The incidence of motor complications (dyskinesia, motor fluctuations), impulsive-compulsive behaviors and adverse events and clinical disability rating scale (UPDRS) scores were evaluated using standard meta-analytic methods. Metaregression was conducted on the incidence of motor complications (dyskinesia) with treatment duration and NEDA dose as covariates.

**Results:**

Patients treated with NEDA had significantly lower UPDRS total scores, motor scores and activity of daily living (ADL) scores than those receiving a placebo (weighted mean difference (WMD) −4.81, 95% CI −6.57 to −3.05; WMD −4.901, 95% CI −7.03 to −2.77; WMD −1.52, 95% CI −2.19 to −0.84, respectively). Patients in the NEDA and NEDA+open Levodopa (LD) groups had lower odds for dyskinesia than patients in the LD group (OR = 0.21, 95% CI: 0.15–0.29; OR = 0.31, 95% CI 0.24–0.42, respectively). Metaregressions indicated that the mean LD dose of the NEDA group increased, and the odds of developing dyskinesia increased (*p* = 0.012). However, the odds of developing dyskinesia in the NEDA group were not related to treatment duration (*p* = 0.308). PD patients treated with NEDA or NEDA+open LD had a lower risk of wearing-off implications than those treated with LD (all *p* < 0.05). No significant difference was found between the NEDA and placebo groups in impulsive-compulsive behavior development (*p* > 0.05). Patients in the NEDA group were more likely to suffer somnolence, edema, constipation, dizziness, hallucinations, nausea and vomiting than those in the placebo or LD group.

**Conclusion:**

NEDA therapy reduces motor symptoms and improves ADLs in early PD. The odds of developing motor complications were lower with NEDA than with LD, and dyskinesia increased with increasing LD equivalent dose and was not influenced by NEDA treatment duration. Therefore, long-term treatment with an appropriate dosage of NEDA might be more suitable than LD for early PD patients.

**Registration:**

PROSPERO CRD42021287172.

## Introduction

Parkinson's disease (PD) is the most common neurodegenerative movement disorder and is characterized by a set of main motor symptoms, including bradykinesia, rigidity and tremor, and additional non-motor symptoms, such as depression, cognitive impairment, insomnia, and fatigue. According to the Global Burden of Disease Study 2016, in 2016, the PD population was almost 6,100,000, and PD had the fastest growth in prevalence, disability, and deaths, leading to poor quality of life in PD patients and an increasing burden on society and families (Tysnes and Storstein, 2017; Collaborators, 2018).

Dopaminergic anti-parkinsonism medicines, especially dopamine agonists (DAs) and levodopa (LD), are still recommended as the first-line treatment for patients with early PD based on the latest NICE guidelines (Rogers et al., 2017). DAs include ergot DAs and non-ergot agonist DAs, but ergot DAs are not recommended in PD treatment because of the risk of fibrotic reactions (Rasmussen et al., 2011). NEDA, mainly pramipexole, ropinirole, rotigotine and piribedil, can effectively reduce the symptoms of PD patients and potentially slow the progression of PD disease (Rogers et al., 2017). However, motor complications develop when using dopaminergic antiparkinsonism agents, which needs to receive more attention and has become one of the most challenging problems for movement disorder doctors. Moreover, comparisons of motor complications between NEDA and LD/placebo are still controversial and lacking. Previous meta-analyses have evaluated the efficacy and safety of DAs in PD and clarified that DAs, including NEDA, had a positive effect in PD (Baker et al., 2009; Zhou et al., 2014b). However, most of the meta-analyses did not assess motor complications (especially dyskinesia) in patients treated with NEDA vs. LD/placebo, so the use of NEDA could not comprehensively evaluated for early PD (Baker et al., 2009; Zhou et al., 2014b). One meta-analysis assessed motor complications in DA interventions. However, this analysis included ergot dopamine agonists that might increase the number of confounding factors in evaluations of NEDA in PD (Chondrogiorgi et al., 2014). Furthermore, no meta-analysis of NEDA has been conducted to examine the relation between the dose of NEDA or the duration of NEDA treatment and the incidence of motor complications (especially dyskinesia).

Therefore, in this study, we conducted a meta-analysis mainly to examine the risk of motor complications between NEDA monotherapy (or plus open-label LD) and either LD or a placebo in early PD. Meta-regression analyses were also performed to evaluate the contribution of the dose of NEDA and the duration of NEDA treatment to development of motor complications. Moreover, clinician-rated disability scales, impulsive-compulsive behaviors and adverse events were also used to fully evaluate the use of NEDA for PD treatment.

## Methods

### Search Strategy

In an attempt to comprehensively identify all eligible studies, four electronic databases, Medline (Ovid), Embase (Ovid), the Cochrane Central Register of Controlled Trials, and Web of Science, were searched from inception through 13 August 2021. The search terms included medical subject headings and free terms regarding intervention treatments (NEDA), disease (PD), and research types (randomized clinical trial designs). The specific search strategies are listed in Supplement 1.

### Inclusion and Exclusion Criteria

Eligible studies were included in the analysis if they met the following criteria: 1) randomized controlled trials (RCTs); 2) study population diagnosed with PD at the early stage and no history of motor complications; 3) intervention treatments contained NEDA vs. placebo/LD (open-label LD was also allowed during the treatment); 4) the absolute number or the incidence of motor complications (dyskinesia or wearing-off) was reported each group or the number or the incidence of impulsive-compulsive behaviors was reported for each group; or clinician-rated disability scale scores [Unified Parkinson's Disease Rating Scale (UPDRS) score] were reported.

Studies were excluded for providing insufficient data or if the outcome measures did not meet the inclusion criteria. Animal trials, clinical protocols, conference abstracts and quasi-RCTs were also excluded.

### Data Extraction and Quality Assessment

Data from and basic information on the eligible studies were extracted by two independent investigators (GHJ, XYS). The following information was extracted from each study: study characteristics (first author, year, design), population (disease at early stage), intervention and control (NEDA with or without open-label LD vs. LD/placebo, mean daily dose of NEDA, frequency, treatment duration), outcome measures (incidence of dyskinesia/wearing-off, impulsive-compulsive behaviors, changes in the UPDRS scores, and incidence of adverse events). The quality of the included studies was evaluated based on the standard criteria of the Cochrane Risk of Bias Tool (Savovic et al., 2014). Two reviewers (TCZ and CDF) conducted the quality assessment independently, and any disagreement was resolved by discussion with a senior investigator (ZML).

### Outcome Measures

The outcome measures mainly included the incidence of motor complications (dyskinesia, motor fluctuations), incidence of impulsive-compulsive behaviors, mean daily dose of NEDA with dyskinesia, duration of treatment with NEDA with dyskinesia, UPDRS scores (II, III, and total) and incidence of adverse events.

### Statistical Analysis

We conducted a meta-analysis of eligible studies by using Stata version 16. The incidences of dyskinesia/wearing-off, impulsive-compulsive behaviors and adverse events were regarded as dichotomous data and are presented as odds ratios (ORs) with 95% CIs. The mean change in the UPDRS scores from baseline was treated as continuous data and is reported as the weighted mean difference (WMD) with a 95% confidence interval (CI). Statistical heterogeneity was estimated using the *I*^2^ statistic; if heterogeneity existed, a random effects model was used. Otherwise, the fixed-effects model was used to pool the results. If the pooled result exhibited clinically relevant heterogeneity, subgroup analysis was also conducted to identify the source of heterogeneity (Cumpston et al., 2019).

Meta-regression analyses were also performed to evaluate the relationship between LD equivalent doses (LEDs) of NEDA and dyskinesia. In addition, treatment duration was included independently as a covariate in the meta-regression to explore potential risk factors for motor complications (dyskinesia).

Finally, a sensitivity analysis was performed by removing one study at a time to estimate the stability of the results. The publication bias of the included studies was examined by Egger's test.

## Results

### Study Identification and Selection

**Figure 1:**
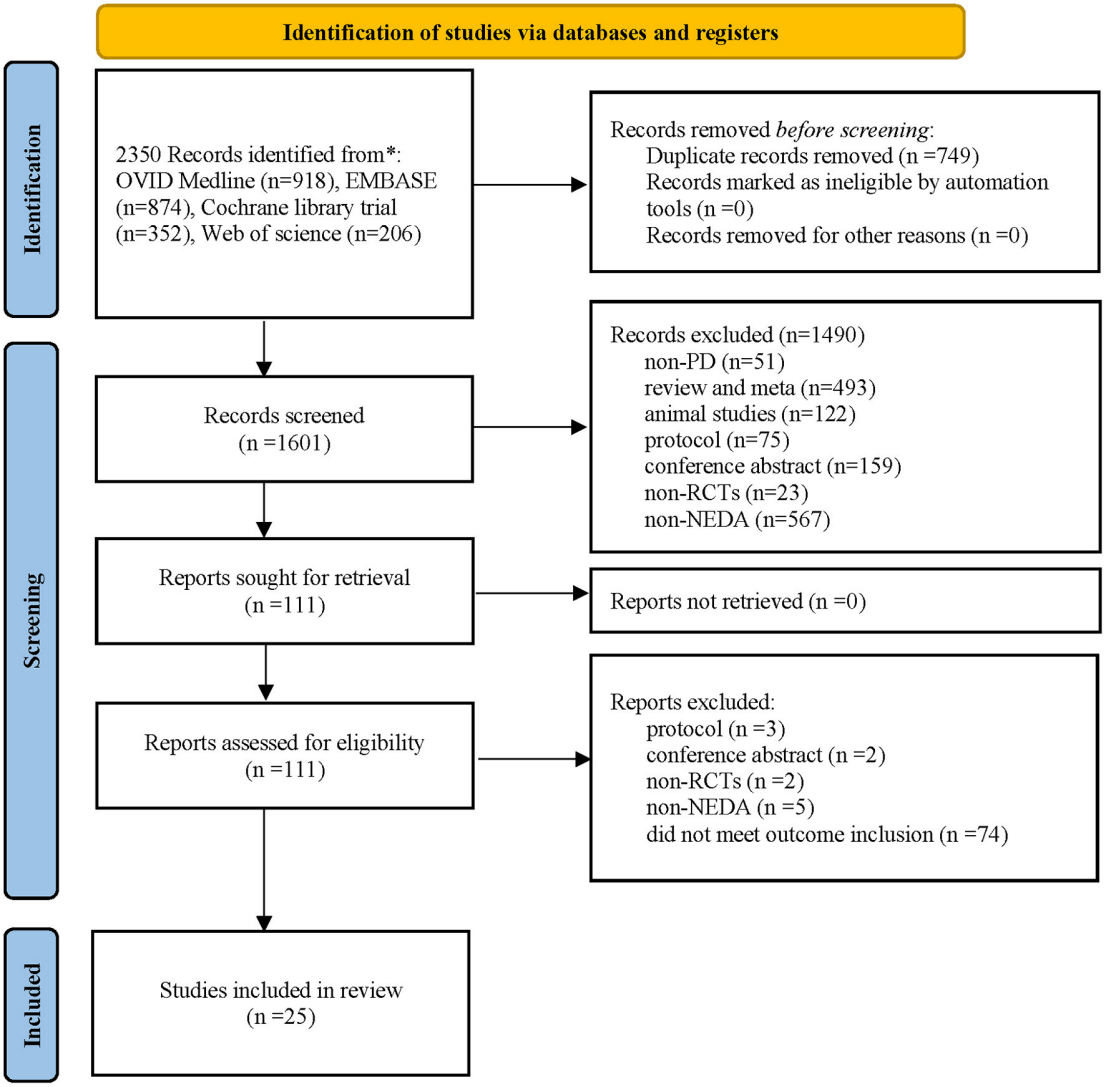
Study flow diagram.

A total of 2,350 potentially eligible publications were identified in the four electronic databases, and 749 duplicate studies were eliminated. A total of 1,601 publications underwent further screening via review of their titles and abstracts. Then, 1,490 publications were excluded, and the remaining 111 studies underwent full-text review. Ultimately, 25 publications involving 6,427 patients met the inclusion criteria and were included in the quantitative meta-analysis [the specific PRISMA 2020 flow diagram (Page et al., 2021) is described in Figure 1].

### Characteristics of the Included Studies

A total of 25 studies involving 6,427 participants who were diagnosed with early PD met the inclusion criteria. The NEDA treatments included in this meta-analysis were pramipexole, ropinirole, rotigotine, and piribedil. Placebo or LD treatment was adopted as the control in the included trials. The length of intervention varied from 9 to 480 weeks; the mean LED in the NEDA group ranged from 150 to 712 mg. The frequency of medicine use ranged from 1 to 3 times per day. Sixteen studies reported the occurrence of dyskinesia as an outcome; 7 studies reported the incidence of motor fluctuations; two studies reported the incidence of impulsive-compulsive behaviors; 12 trials recorded the mean daily dose of NEDA with dyskinesia; 17 studies used the UPDRS score (II, III, and total) as an outcome measure; and 22 studies recorded the incidence of adverse events (see Supplementary Table 1).

### Quality of the Included Studies

Sixteen studies (64%) reported the methods of random sequence generation, and 68% had a low risk for allocation concealment. Nineteen studies (76%) used blinding methods for participants and personnel. Eighteen studies had a low risk bias for blinding the outcome assessment. Twenty-five trials had a low risk of attrition bias and reporting bias. Overall, nineteen studies (76%) were regarded as having a low risk of poor methodological quality (Supplementary Figures 1, 2).

### Analysis of Outcomes

#### Clinical Disability Rating Scales (UPDRS)

**Figure 2:**
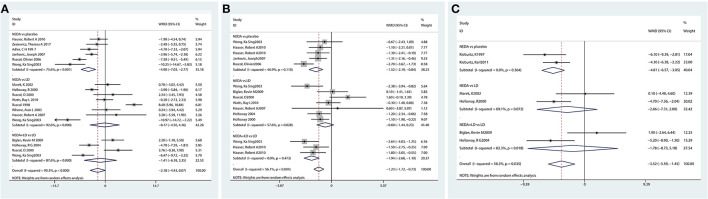
Effect of NEDA on Unified Parkinson's Disease Rating Scale scores. **(A)** Effect of NEDA on Unified Parkinson's Disease Rating Scale motor score. **(B)** Effect of NEDA on Unified Parkinson's Disease Rating Scale Activities of Daily Living score. **(C)** Effect of NEDA on Unified Parkinson's Disease Rating Total Scale. CI, confidence interval; df, degrees of freedom; NEDA, non-ergot dopamine agonist; PD, Parkinson's disease; SD, standard deviation.

A total of 15 studies examined NEDA (NEDA+open LD) vs. a placebo/LD. In terms of the UPDRS motor subscale, patients who received NEDA had a significant reduction in motor scores compared with those receiving placebo (WMD −4.901, 95% CI −7.03 to −2.77; *p* < 0.01). However, no significant reduction in motor scores was seen between NEDA and LD or between NEDA+open LD and LD (WMD −0.171, 95% CI −4.50 to 4.16; WMD −1.42, 95% CI −6.18 to 3.35; all *p* > 0.05). The pooled results for the UPDRS motor scores exhibited significant heterogeneity among the three drug class subgroups (*p* < 0.001, *I*^2^ = 90.3%). The use of various drug interventions and control groups might be the sources of heterogeneity (Figure 2A).

A total of 10 trials assessed the UPDRS ADL subscale. Compared with a placebo, NEDA were associated with a significant improvement in the UPDRS-ADL score (WMD −1.52, 95% CI −2.19 to −0.84; *p* < 0.01). Patients who received NEDA + open LD had significantly reduced UPDRS-ADL scores compared with those receiving LD alone (WMD −1.94, 95% CI −2.68 to −1.19, *p* < 0.05). However, there was no significant difference in UPDRS-ADL score between NEDA and LD (WMD −0.60, 95% CI −1.44 to 0.23, *p* > 0.05). The heterogeneity among subgroup drug classes was significantly different (*p* < 0.01, *I*^2^ = 56.1%) (Figure 2B).

Six studies reported the UPDRS total score, and the NEDA group had significantly reduced UPDRS total scores compared with the placebo group (WMD −4.81, 95% CI −6.57 to −3.05; *p* < 0.01). No significant differences in UPDRS-total were seen between NEDA and LD or between NEDA+open LD and LD (WMD −2.66, 95% CI −7.31 to 2; WMD −1.78, 95% CI −8.73 to 5.18; all *p* > 0.05). Subgroup heterogeneity also existed among different drug classes (*p* = 0.035, *I*^2^ = 58.3%) (Figure 2C).

### Motor Complications

#### Dyskinesia

##### Incidence of Dyskinesia

**Figure 3:**
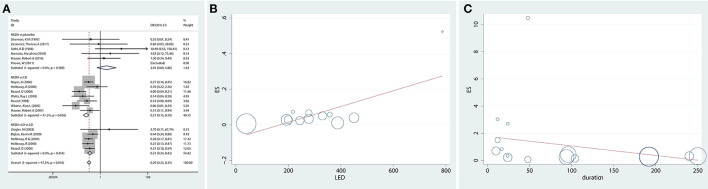
Incidence of dyskinesia and potential risk factors for dyskinesia in trials of NEDA. **(A)** Forest plot for incidence of dyskinesia in trials of NEDA. **(B)** Meta-regression plot representing the association between dyskinesia and the mean LED of NEDA. **(C)** Meta-regression plot representing the association between dyskinesia and NEDA treatment duration. Circles represent individual trials. The size of the circles is proportional to the inverse of the variance in the incidence of dyskinesia found in that trial. OR, odds ratio; CI, confidence interval; LD, levodopa; NEDA, non-ergot dopamine agonist.

A total of 16 studies reported the incidence of dyskinesia for NEDA compared with a placebo or LD. The results revealed that patients in the NEDA group did not have significantly greater odds of developing dyskinesia than patients in the placebo group (OR = 2.01, 95% CI 0.69–5.86). Patients in the NEDA and NEDA+open LD groups had lower odds (79 and 69%, respectively) than patients in the LD group (OR = 0.21, 95% CI 0.15–0.29; OR = 0.31, 95% CI 0.24–0.42, respectively). Each subgroup had low heterogeneity (*p* = 0.589; *p* = 0.454; *p* = 0.056, respectively), while heterogeneity existed among the various subgroups (*p* = 0.016, *I*^2^ =47.3%) (Figure 3A).

##### Association Between Dyskinesia and the Mean LED of NEDA (Meta-Regression)

Twelve studies reported the dose of NEDA and converted them to LEDs of NEDA. To further explore the relationship between the mean LED of NEDA and dyskinesia, we used meta-regression, and the results showed that as the mean LD dose of NEDA increased, the odds of developing dyskinesia increased [exp(b), SE = 1.00, 0.00014, *p* = 0.012) (Figure 3B).

##### Association Between Dyskinesia and NEDA Treatment Duration (Meta-Regression)

We also examined the association between dyskinesia and NEDA treatment duration, and the results indicated that the odds of developing dyskinesia in the NEDA group, compared with that in the placebo and LD groups, were not related to treatment duration [exp(b), SE = 0.99, 0.0066, *p* = 0.308] (Figure 3C).

#### Incidence of Wearing-Off

**Figure 4:**
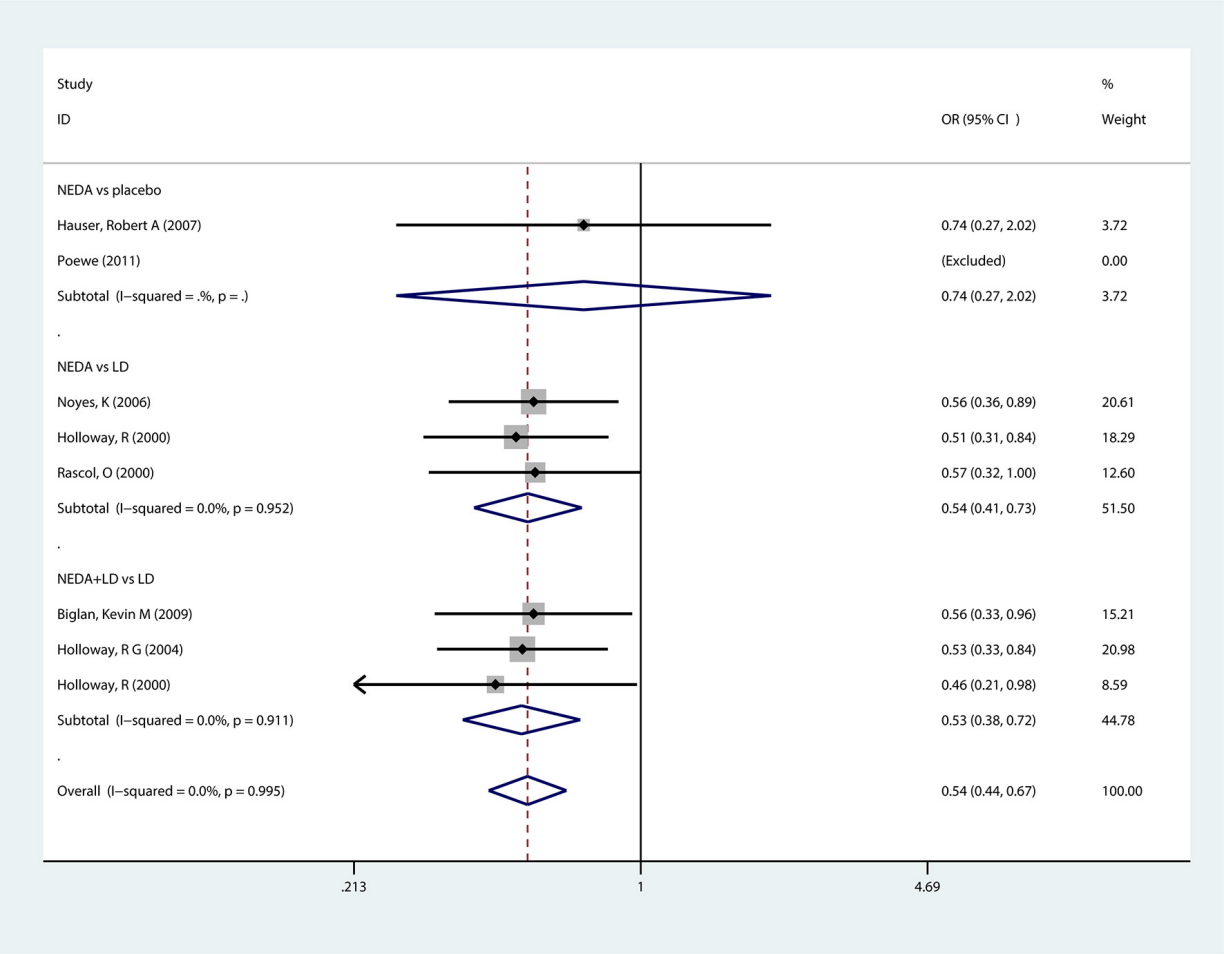
Forest plot for incidence of wearing-off in trials of NEDA. OR, odds ratio; CI, confidence interval; LD, levodopa; NEDA, non-ergot dopamine agonist.

Seven trials reported motor implications (wearing-off) as an outcome measure. The results showed that PD patients who received NEDA or NEDA+open LD had (46%, 47%) lower odds of developing wearing-off implications than those in the LD group (OR = 0.54, 95% CI 0.41–0.73; OR = 0.53, 95% CI 0.38–0.72, respectively). However, there was no significant difference in the incidence of wearing-off outcomes between NEDA and a placebo (OR = 0.74, 95% CI 0.27–2.02). Each subgroup and all groups had low heterogeneity (*p* = 0.952; *p* = 0.911; respectively) (Figure 4).

#### Impulsive-Compulsive Behaviors

**Figure 5:**
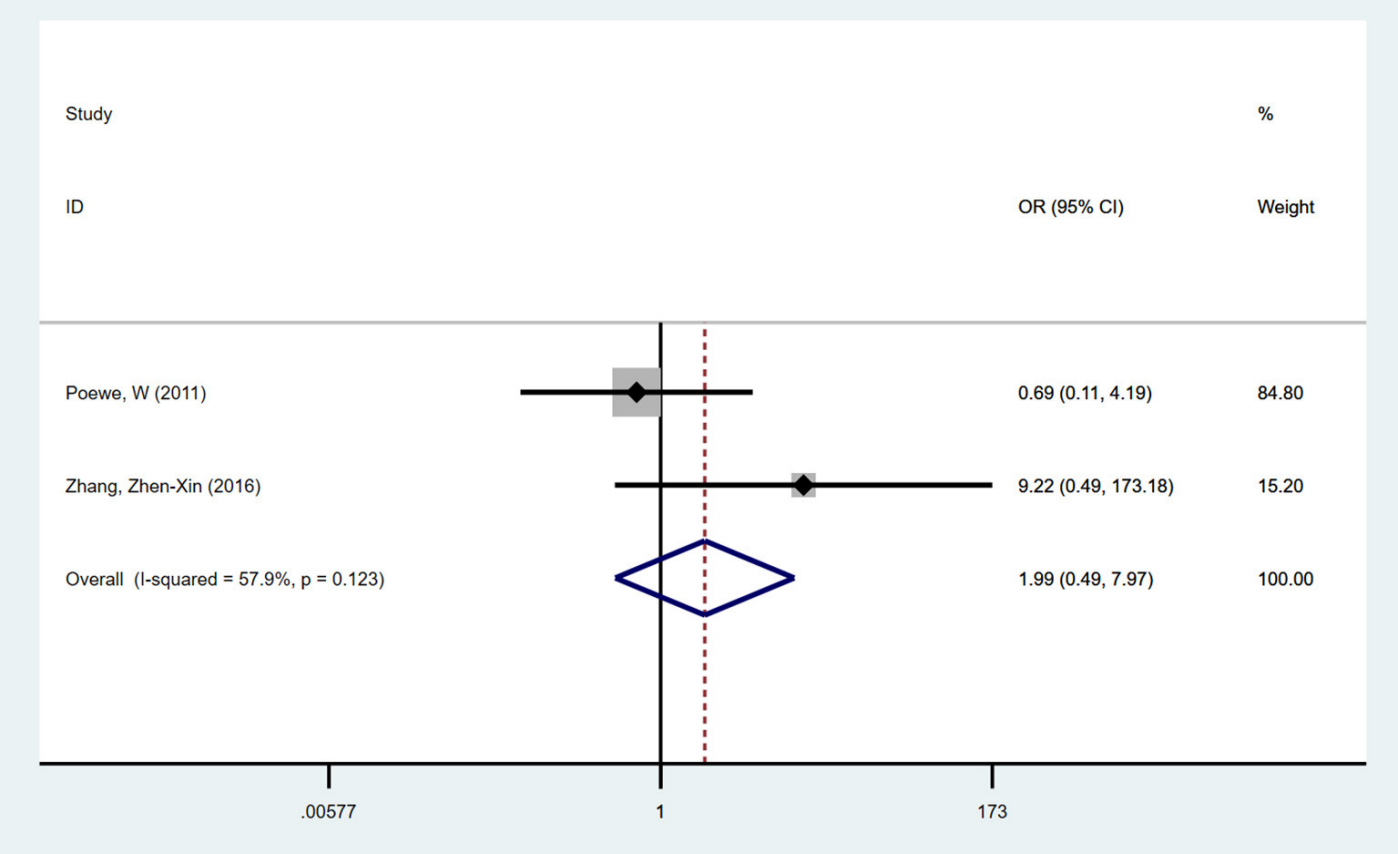
Forest plot for incidence of impulse compulsive behaviors in trials of NEDA. OR, odds ratio; CI, confidence interval; LD, levodopa; NEDA, non-ergot dopamine agonist.

A total of two trials examined impulsive-compulsive behaviors with NEDA vs. a placebo. No significant difference was seen in the development of impulsive-compulsive behaviors in the NEDA group compared with the placebo group (OR = 1.986, 95% CI 0.495, 7.971). No heterogeneity existed among the two trials (*p* = 0.123, *I*^2^ = 57.9%) (Figure 5).

#### Adverse Events

A total of 22 studies that reported 18 types of adverse events (each event was reported in at least three studies) were analyzed in this study. The pooled incidences of eighteen types of adverse events, including vomiting, somnolence, nausea, and edema, are shown in Table 1. Patients who took NEDA were more likely to suffer somnolence, edema, constipation, dizziness, hallucinations, nausea, and vomiting than those in the placebo or LD group.

**Table 1:** The adverse events of NEDA in Parkinson's disease.

**Adverse events**	**Comparisons**	**OR (95%CI)**	***P***	***I*^2^%**
Abdominal pain	NEDA vs. LD	0.853 (0.429, 1.693)	0.649	0.00
Arthralgia	NEDA vs. LD	1.027 (0.592, 1.784)	0.924	0.00
Back pain	NEDA vs. LD	1.370 (0.709, 2.646)	0.349	0.0
	NEDA vs. placebo	1.795 (0.559, 5.764)	0.325	0.0
Constipation	NEDA vs. placebo	2.038 (1.220, 3.406)	0.007	37.2
	NEDA vs. LD	4.607 (2.237, 9.488)	0.000	0.0
	NEDA+LD vs. LD	0.753 (0.265, 2.142)	0.595	82.5
	NEDA+LD vs. placebo	1.362 (0.463, 4.003)	0.575	53.8
Diarrhea	NEDA vs. placebo	1.121 (0.177, 7.113)	0.904	71.7
Dizziness	NEDA vs. LD	1.161 (0.834, 1.617)	0.377	0.0
	NEDA vs. placebo	1.636 (1.215, 2.204)	0.001	0.0
	NEDA+LD vs. LD	1.216 (0.818, 1.807)	0.334	4.6
	NEDA+LD vs. placebo	1.441 (0.721, 2.879)	0.301	0.0
Dyspepsia	NEDA vs. LD	1.447 (0.415, 5.041)	0.562	63.70
Edema	NEDA vs. LD	3.108 (1.360, 7.103)	0.007	0.00
	NEDA+LD vs. LD	0.973 (0.053, 17.771)	0.985	98.10
Fatigue	NEDA vs. placebo	1.097 (0.725, 1.660)	0.661	27.2
	NEDA vs. LD	1.885 (0.929, 3.827)	0.079	0.0
Hallucination	NEDA vs. placebo	4.671 (1.712, 12.748)	0.003	24.3
	NEDA vs. LD	2.269 (1.241, 4.148)	0.008	0.0
Headache	NEDA vs. LD	1.009 (0.629, 1.617)	0.972	0.0
	NEDA+LD vs. LD	1.083 (0.691, 1.697)	0.728	50.0
	NEDA vs. placebo	1.210 (0.835, 1.754)	0.315	3.9
Insomnia	NEDA vs. placebo	1.463 (0.823, 2.603)	0.195	46.2
	NEDA vs. LD	1.442 (0.955, 2.176)	0.082	0.0
	NEDA+LD vs. LD	0.929 (0.523, 1.653)	0.803	50.6
	NEDA+LD vs. placebo	1.867 (0.804, 4.339)	0.147	0.0
Nausea	NEDA vs. placebo	2.520 (1.992, 3.186)	0.000	36.5
	NEDA vs. LD	1.301 (1.004, 1.685)	0.046	47.4
Peripheral edema	NEDA vs. placebo	2.548 (1.062, 6.113)	0.036	0.0
	NEDA vs. LD	4.351 (2.169, 8.729)	0.000	0.0
	NEDA+LD vs. LD	1.487 (0.122, 18.098)	0.756	82.3
	NEDA+LD vs. placebo	1.515 (0.593, 3.870)	0.386	0.0
Somnolence	NEDA vs. placebo	4.214 (2.769, 6.412)	0.000	60.0
	NEDA vs. LD	2.190 (1.263, 3.800)	0.005	49.8
	NEDA+LD vs. LD	1.584 (0.735, 3.415)	0.240	87.1
Tremor	NEDA vs. placebo	0.624 (0.222, 1.752)	0.371	38.0
	NEDA vs. LD	1.342 (0.224, 8.030)	0.747	75.3
Vomiting	NEDA vs. placebo	3.848 (1.633, 9.066)	0.002	0.0
URTI	NEDA vs. placebo	0.882 (0.525, 1.482)	0.636	0.0

*CI, confidence interval; NEDA, non-ergot dopamine agonist; URTI, upper respiratory tract infection; N/A, not available*.

### Sensitivity Analysis

We conducted a sensitivity analysis by excluding one study at a time to examine the robustness of the pooled results. The analysis showed that the pooled results for the incidence of dyskinesia/motor fluctuations, impulsive-compulsive behaviors and clinical disability rating scale outcomes were stable and not influenced by the individual data when excluding one study at a time (Supplementary Figure 3).

### Publication Bias

No significant publication bias was seen in the included studies based on Egger's test or Begg's test for different outcome measures [incidence of dyskinesia, Egger's test *p* = 0.161; incidence of motor fluctuations, Egg's test *p* = 0.471; UPDRS-II (ADL), Egger's test *p* = 0.218; UPDRS-motor, Egger's test *p* = 0.503; UPDRS-total, Egger's test *p* = 0.214, Supplementary Table 2].

## Discussion

### Summary of Findings

We conducted a meta-analysis of 25 trials with 6,427 participants. Motor complications (incidence of dyskinesia/wearing-off) and impulsive-compulsive behaviors were the primary outcomes used to assess the safety of the use of NEDA for PD. Moreover, clinical disability rating scales and adverse events related to NEDA use were also examined to comprehensively evaluate the effect of NEDA in patients with PD. After analyzing the pooled results, we arrived at several conclusions.

First, our pooled results indicated that NEDA were associated with significant improvement in the UPDRS-motor, UPDRS-ADL and UPDRS-total scale scores, compared with those for the placebo group. No significant differences were seen between the NEDA and LD groups on the UPDRS scales. These results demonstrated that NEDA could ameliorate motor function and ADLs in PD to some extent, and the treatment effect of NEDA was almost equal to that of LD. Previous studies reported similar results, i.e., that the use of NEDA reduced clinical disability rating scale (UPDRS) scores and improved motor ability and ADLs in PD patients (Zhou et al., 2014a; Cerri and Blandini, 2020; Poewe and Mahlknecht, 2020). However, most studies have demonstrated that LD is the most efficacious medication in the treatment of motor symptoms and might provide better symptom control than NEDA (Isaacson and Hauser, 2009; Sy and Fernandez, 2020).

NEDA were beneficial for ameliorating PD symptoms. The safety considerations and side effects of NEDA need to be considered to fully understand the value of NEDA for PD patients and help doctors make optimal decisions. Our pooled results demonstrated that NEDA in general have a low risk of causing dyskinesia, which was similar to the placebo group. However, NEDA are associated with a lower incidence of dyskinesia than LD treatment. Dyskinesia is a common motor implication that occurs when taking dopaminergic antiparkinsonism medicine. Both NEDA and LD were beneficial for alleviating Parkinsonian motor symptoms, and LD was regarded as more efficacious than NEDA in symptom improvement; however, its long-term use was more strongly associated with the development of motor complications (Stowe et al., 2008; Isaacson and Hauser, 2009; Fox et al., 2018). Overall, NEDA might be the safe, optimal treatment option for PD patients in the early stage and possibly for the long term. The incidence of dyskinesia induced by NEDA is still controversial among current studies. However, most studies suggest that NEDA use is associated with a lower odds of developing dyskinesia than LD use, which is consistent with our results (Tambasco et al., 2012; Stathis et al., 2015; Poewe and Mahlknecht, 2020). In addition, we analyzed the relationship between the dose of NEDA and the incidence of dyskinesia using metaregression, and the results suggested that the incidence of dyskinesia significantly increased with increasing mean dose of NEDA, which meant that the mean LED was a potential risk factor for dyskinesia. Previous studies also indicated that increasing the LED would increase the odds of developing dyskinesia (Walker et al., 2011; Warren Olanow et al., 2013; Cabreira et al., 2019; Hong et al., 2020). Therefore, the dose of NEDA needs to balance the alleviation of PD symptoms and the potential risk of dyskinesia. However, we also found that the odds of dyskinesia did not increase with the duration of NEDA treatment, which was in contrast to findings on the long-term use of LD and supports that the cumulative dose of NEDA was safe (Kondo, 2002; Pilleri and Antonini, 2015; Guttler et al., 2021).

The results demonstrated that PD patients who received NEDA had a lower risk of wearing-off motor implications than patients who received LD. Previous studies drew a similar conclusion that PD patients who receive NEDA long-term have a potential risk of suffering wearing-off implications when maintaining symptomatic control but it is greater than that of patients who use LD (Schrag et al., 2007; Baker et al., 2009; Jenner, 2013).

A few studies reported impulsive-compulsive behaviors while taking NEDA, and the results indicated that early PD patients taking NEDA therapies did not have significantly different odds of impulsive-compulsive behaviors compared with those taking a placebo. However, the potential risk of developing impulsive-compulsive behaviors from NEDA use still exists and needs attention. Recent studies have also demonstrated that impulsive-compulsive behaviors might easily occur when patients use NEDA long term or at higher doses (Napier et al., 2020; Cao et al., 2021).

Furthermore, we examined the adverse events reported in at least three trials, and the results indicated that somnolence, edema, constipation, dizziness, hallucinations, nausea, and vomiting were the most frequent adverse events occurring in early PD patients treated with NEDA. However, none of these adverse events were severe, and they resolved after adjusting the dose of NEDA.

Regarding the quality of the included studies, 76% were regarded as high-quality studies, which indicated that the evidence that we analyzed was robust.

### Findings in Relation to Previous Reviews

To our knowledge, this meta-analysis was the first study that comprehensively examined the efficacy and safety of NEDA in early stage PD patients. We focused mainly on the evaluation of motor implications, especially dyskinesia induced by NEDA. We also explored potential risk factors (dose of LED, duration of NEDA treatment) that might influence the incidence of motor complications. One previous study conducted a meta-analysis to examine the efficacy of NEDA in early and later PD patients. The pooled results regarding the effects of NEDA on motor function were consistent with our results. However, that study mainly focused on motor function improvements, and potential risk factors for motor complications were not analyzed (Zhou et al., 2014b). The side effects of NEDA are still essential for determining how NEDA are applied. Another study assessed the incidence of and risk factors for dyskinesia induced by DAs (including NEDA and EDAs) in PD treatment. This study indicated that DAs decreased the odds of developing dyskinesia compared with LD treatment, which was similar to our result that indicates the positive effect of NEDA. However, this meta-analysis included most EDAs (a class of medicines that might easily cause fibroblasts and are not recommended for use as first-line medicines), which might weaken the strength of evidence and increase the number of confounding factors in analyzing the effect and safety of NEDA (Chondrogiorgi et al., 2014). Therefore, in this meta-analysis, we comprehensively assessed the incidence of motor implications for NEDA (or plus supplemental LD) and LD (or placebo) and determined whether potential risk factors, including dose response and dose accumulation, might influence the incidence of dyskinesia in PD patients.

### Implications for Clinical Practice

Several implications for clinical practice were obtained from our analysis. First, clinical doctors could suggest that patients with early PD use NEDA as an evidence-based alternative for ameliorating motor symptoms and improving ADLs. Second, considering the low odds of developing motor implications, NEDA are recommended for long-term treatment for early PD patients, especially patients who do not urgently require significant alleviation of motor symptoms. Third, healthcare workers still need to control the dose of NEDA because the risk of dyskinesia increases with increasing dose of NEDA. However, the incidence of dyskinesia was not influenced by NEDA treatment duration, meaning that NEDA might be more suitable for long-term treatment. Last, doctors still need to pay attention to the side effects of NEDA, and alternative options are needed when serious side effects occur.

### Limitations

There were several limitations in this meta-analysis, and the above findings should be interpreted with caution. Few studies have reported impulsive-compulsive behaviors during NEDA treatment, and it is difficult to provide comprehensive evidence to examine impulsive-compulsive behaviors associated with NEDA use. Moreover, a part of the DA monotherapy group received open LD during the trial or follow-up period, which might confound the analysis of the incidence of motor complications and side effects induced by NEDA monotherapy. Therefore, we excluded patients treated with NEDA + open LD from the NEDA monotherapy group and classified them as the NEDA + open LD group. However, excluding these studies from the NEDA monotherapy group might have removed some important data concerning NEDA monotherapy, which increased the incomplete reporting bias and weakened the strength of the evidence. Additionally, in this study, we focused mainly on exploring potential risk factors influencing the occurrence, rather than the severity, of motor complications. The factors that influence the severity of motor complications should be given more attention in future studies.

## Conclusion

In conclusion, NEDA therapy effectively reduced motor symptoms and ameliorated ADLs in the early stage of PD. However, the incidence of motor implications, especially dyskinesia, was still influenced by NEDA use. Dyskinesia increased with increasing LED and was not influenced by the duration of NEDA treatment. Although a high dose of NEDA might increase the odds of developing motor complications, the odds were still lower than those associated with LD use. Considering the lower risk of motor complications and smaller dose accumulation effect, NEDA might be more suitable for early PD patients undergoing long-term treatment.

## Data Availability Statement

The original contributions presented in the study are included in the article/Supplementary Material, further inquiries can be directed to the corresponding authors.

## Author Contributions

MZ and DC designed the study and revised the manuscript for important intellectual content. YX, HG, LL, and XL acquired the data. CW and CT analyzed and interpreted the data. CW drafted the manuscript. All authors read and approved the final manuscript.

## Funding

This study was supported by the China Postdoctoral Science Foundation (No. 2020M682684); Natural Science Foundation of Guangdong Province (No. 2020A151501325); the Project of Administration of Traditional Chinese Medicine of Guangdong Province (No. 20203010); State Administration of Traditional Chinese Medicine of the People's Republic of China (No. GZYFJS-2019001); Sanming Project of Medicine in Shenzhen (No. SZZYSM202106009); and Bao'an TCM Development Foundation (No. 2020KJCX-KTYJ-131).

## Conflict of Interest

The authors declare that the research was conducted in the absence of any commercial or financial relationships that could be construed as a potential conflict of interest.

## Publisher's Note

All claims expressed in this article are solely those of the authors and do not necessarily represent those of their affiliated organizations, or those of the publisher, the editors and the reviewers. Any product that may be evaluated in this article, or claim that may be made by its manufacturer, is not guaranteed or endorsed by the publisher.
