# Supplementary material for: The Comparative Efficacy of Non-ergot Dopamine Agonist and Potential Risk Factors for Motor Complications and Side Effects From NEDA Use in Early Parkinson's Disease: Evidence From Clinical Trials
Source: Front Aging Neurosci. 2022 Apr 22;14:831884. doi: 10.3389/fnagi.2022.831884 (PMC9074827; doi:10.3389/fnagi.2022.831884)
Supplement: Supplementary file 1 [file Data_Sheet_1.docx]

**Supplementary Online Content**

[Supplementary 1. Search strategy for each database 2](#_Toc95042924)

[Supplementary Table 1. Detailed information of included studies. 6](#_Toc95042925)

[Supplementary Table 2. Tests for Publication Bias 9](#_Toc95042926)

[Supplementary Figure 1. Risk of bias graph 10](#_Toc95042927)

[Supplementary Figure 2. Risk of bias summary 11](#_Toc95042928)

[Supplementary Figure 3 Sensitivity analysis 12](#_Toc95042929)

[Supplementary references 15](#_Toc95042930)

**Supplementary 1. Search strategy for each database**

**MEDLINE (OVID)**

1 Parkinson.tw. 20649

2 Parkinson$.tw. 121036

3 (PD or IPD).tw. 144968

4 (Parkinson$ adj5 Diseas$).tw. 100258

5 exp Parkinson Disease/ 68260

6 1 or 2 or 3 or 4 or 5 226474

7 exp Dopamine Agonists/ 32310

8 dopamin* agonist*.tw. 9083

9 exp pramipexole/ 974

10 pramipexol*.tw. 1315

11 ropinirol*.tw. 822

12 ropinirole.mp. 956

13 rotigotine.mp. 584

14 rotigotin*.tw. 469

15 piribedil.mp. 561

16 piribedil*.tw. 433

17 7 or 8 or 9 or 10 or 11 or 12 or 13 or 14 or 15 or 16 36764

18 (early or "de novo" or untreated).mp. 1860671

19 randomized controlled trial.pt. 520899

20 controlled clinical trial.pt. 94019

21 randomized.ab. 508610

22 placebo.ab. 215106

23 drug therapy.fs. 2269500

24 randomly.ab. 350353

25 trial.ab. 539638

26 groups.ab. 2150347

27 19 or 20 or 21 or 22 or 23 or 24 or 25 or 26 4902893

28 6 and 17 and 18 and 27 918

**Embase (OVID)**

1 Parkinson.tw. 28499

2 Parkinson$.tw. 171753

3 (PD or IPD).tw. 248239

4 (Parkinson$ adj5 Diseas$).tw. 145887

5 exp Parkinson Disease/ 160151

6 1 or 2 or 3 or 4 or 5 373725

7 exp Dopamine Agonists/ 211644

8 dopamin* agonist*.tw. 12523

9 exp pramipexole/ 7126

10 pramipexol*.tw. 2289

11 ropinirol*.tw. 1455

12 ropinirole.mp. 5323

13 rotigotine.mp. 2442

14 rotigotin*.tw. 892

15 piribedil.mp. 1823

16 piribedil*.tw. 637

17 7 or 8 or 9 or 10 or 11 or 12 or 13 or 14 or 15 or 16 212933

18 (early or "de novo" or untreated).mp. 2507711

19 randomized controlled trial/ 641038

20 controlled clinical trial/ 465718

21 random$.tw. 1624031

22 placebo.ab. 309864

23 trial.ab. 783523

24 19 or 20 or 21 or 22 or 23 2302506

25 6 and 17 and 18 and 24 874

**Cochrane Central Register of Controlled Trials**

#1 Parkinsonian Disorders 498

#2 (parkinson*) 11714

#3 (PD or IPD) 42094

#4 #1 or #2 or #3 48456

#5 Dopamine Agonists 1345

#6 dopamin* agonist* 2533

#7 pramipexole 504

#8 pramipexol* 511

#9 ropinirole 327

#10 ropinirol* 330

#11 rotigotine 278

#12 rotigotin* 279

#13 piribedil 121

#14 piribedil* 121

#15 #5 or #6 or #7 or #8 or #9 or #10 or #11 or #12 or #13 or #14 3182

#16 (early or "de novo" or untreated) 145615

#17 #4 and #15 and #16 443

Trials 352

**Web of science**

Indexes=SCI-EXPANDED, SSCI, A&HCI, ESCI, Timespan=All years

#1 TS=(Parkinson*) OR TI=(Parkinson*)

#2 TS=(pramipexole) OR TS=(ropinirole) OR TS=(rotigotine) OR TS=( piribedil)

#3 TS=(early) OR TS=( "de novo") OR TS=(untreated)

#4 TS=(trial) OR TI=(trial) OR TI=(random*) OR TI=(placebo) OR TI=(control*)

#5 #1 and #2 and #3 and #4

# Supplementary Table 1. Detailed information of included studies.

| **Author** | **year** | **Country** | **Study Population** | **Age(y, mean±SD)** | **Gender, N male (%)** | **disease duration（yr）mean (SD)** | **Follow up period** | **Intervention** | **Control** | **Outcomes measure** | **Adverse events** |
| --- | --- | --- | --- | --- | --- | --- | --- | --- | --- | --- | --- |
| Shannon (Shannon et al., 1997) | 1997 | USA | Parkinson disease | NA | NA | NA | 24w | Pramipexole | placebo | incidence of dyskinesia | Nausea; Insomnia; Constipation; Somnolence; Fatigue; Orthostatic blood pressure; Hallucinations; Peripheral edema; Asthenia; Vomiting |
|  |  |  |  |  |  |  |  | dose:0.125-1.5mg |  |  |  |
|  |  |  |  |  |  |  |  | freq: 3 times per day |  |  |  |
|  |  |  |  |  |  |  |  | length of intervention:24weeks |  |  |  |
| Poewe (Poewe et al., 2011) | 2011 | Austria | Parkinson disease | GROUP 1: 61.3 (9.8) GROUP 2:62.0 (9.6) | GROUP 1: 127(57.0)  GROUP 2: 51(49.5) | GROUP 1: 1.0 (1.2) GROUP 2:0.9 (1.0) | NA | Pramipexole ER; PramipexoleIR | placebo | incidence of dyskinesia;impulse compulsive behaviorsincidence of motor fluctuations | Somnolence; Nausea; Constipation; Dizziness; Dry mouth |
|  |  |  |  |  |  |  |  | dose: Pramipexole ER 0.375 -4.5mg;Pramipexole IR 0.125-1.5mg |  |  |  |
|  |  |  |  |  |  |  |  | freq: Pramipexole ER 1 times per day;Pramipexole IR 3 times per day |  |  |  |
|  |  |  |  |  |  |  |  | length of intervention:33 weeks |  |  |  |
| Hauser(Hauser et al., 2010) | 2010 | USA | Parkinson disease | GROUP1:61.6 (9.4) GROUP 2:63.2 (8.7) | GROUP1:62(58.5)  GROUP 2: 44(23) | GROUP1:1.1 (1.3) GROUP 2: 0.8 (1.1) | NA | Pramipexole ER+open LD;Pramipexole IR+open LD | Placebo;Placebo+ open LD | Levodopa equivalent doses ;UPDRS III-motor;UPDRS-II(ADL) | Somnolence; Nausea; Constipation; Fatigue |
|  |  |  |  |  |  |  |  | dose:Pramipexole ER 0.375-4.5mg;Pramipexole IR 0.125-1.5mg |  |  |  |
|  |  |  |  |  |  |  |  | freq: Pramipexole ER 1 times per day;Pramipexole IR 3 times per day |  |  |  |
|  |  |  |  |  |  |  |  | length of intervention:18 weeks |  |  |  |
| Zesiewicz (Zesiewicz et al., 2017) | 2017 | USA | Parkinson disease | GROUP 1:62.1(11.38) GROUP 2: 63.1(8.82) | GROUP 1:25(61)  GROUP 2: 23(57) | NA | 2w | Ropinirole PR | placebo | incidence of dyskinesia;Levodopa equivalent doses ;UPDRS III-motor | Nausea; Somnolence; Headache; Dizziness; Sudden onset of sleep; Hypertension; Vomiting; Back pain |
|  |  |  |  |  |  |  |  | dose:2mg;4mg;8mg;12mg;24mg |  |  |  |
|  |  |  |  |  |  |  |  | freq: 1 times per day |  |  |  |
|  |  |  |  |  |  |  |  | length of intervention:17 weeks |  |  |  |
| Sethi (Sethi, 1998) | 1998 | USA | Parkinson disease | GROUP 1:61.6(11.1) GROUP 2: 62.1(10.8) | GROUP 1:44(62.9)  GROUP 2: 48(62.3) | GROUP 1:1.96(1.57) GROUP 2: 1.88(1.63) | 1.4w | Ropinirole | placebo | incidence of dyskinesia;Levodopa equivalent doses | Somnolence; Dizziness; Arthralgia; Upper respiratory; Headache; Nausea;Myalgia; Hallucination; Tremor; Insomnia; Injury; Depression; Vomiting;Fatigue; Hyperkinesia; Pain |
|  |  |  |  |  |  |  |  | dose:1-8mg |  |  |  |
|  |  |  |  |  |  |  |  | freq: 3 times per day |  |  |  |
|  |  |  |  |  |  |  |  | length of intervention:48weeks |  |  |  |
| Nomoto (Nomoto et al., 2018) | 2018 | Japan | Parkinson disease | GROUP 1:65.3(8.12) GROUP 2: 65.9(8.12) | GROUP 1:34(37.8)  GROUP 2: 39(43.3) | GROUP 1:1.97(1.78) GROUP 2: 1.89(1.91) | NA | Rotigotine | placebo | incidence of dyskinesia;Levodopa equivalent doses | Nasopharyngitis; Nausea; Dyskinesia; Somnolence; Visual hallucination; Vomiting; Contusion; Loss of appetite; Blood creatinine phosphokinase increase；Application site pruritus;dizziness;Fall; Orthostatic hypotension; Hallucinate; Auditory hallucination; Delusion; Sudden onset of sleep; Constipation; insomnia; Back pain; Diarrhea; Weight loss; Peripheral edema; Hypokalemia |
|  |  |  |  |  |  |  |  | dose:2-16mg |  |  |  |
|  |  |  |  |  |  |  |  | freq: 24h |  |  |  |
|  |  |  |  |  |  |  |  | length of intervention:12 weeks |  |  |  |
| Hauser (Hauser et al., 2016) | 2016 | USA | Parkinson disease | GROUP 1:68.1（10.5) GROUP 2: 69.0 (11.7) | GROUP 1:27 (65.9)  GROUP 2: 22 (55.0) | GROUP 1:4.9 (4.0) GROUP 2: 3.7 (3.7) | 4w | Rotigotine | placebo | incidence of dyskinesia;Levodopa equivalent doses | Application site reactions; Nausea ; Somnolence ; Depression ; Fall ; Constipation ; Peripheral edema ; Headache ; Dyskinesia ; Fatigue ; Dry mouth ; Insomnia; Suicidal ideation ; Rash ; Tremor |
|  |  |  |  |  |  |  |  | dose:2-6mg;2-8mg |  |  |  |
|  |  |  |  |  |  |  |  | freq: 24h |  |  |  |
|  |  |  |  |  |  |  |  | length of intervention:29 weeks |  |  |  |
| Ziegler (Ziegler et al., 2003) | 2003 | France | Parkinson disease | GROUP 1:63.4 (7.3) GROUP 2: 64.8 (7.6) | GROUP 1:40 (66)  GROUP 2: 28 (52) | GROUP 1:4.6 (2.75) GROUP 2: 4 (2.33) | 3m | Piribedil +LD | Placebo+ LD | incidence of dyskinesia | Gastrointestinal; symptoms; hypotension; Delirium; Dizziness; Vasodilation hands; Dyestonia; Headche |
|  |  |  |  |  |  |  |  | dose:50-150mg |  |  |  |
|  |  |  |  |  |  |  |  | freq:3 times per day |  |  |  |
|  |  |  |  |  |  |  |  | length of intervention:24 weeks |  |  |  |
| Biglan (Parkinson Study Group, 2009) | 2009 | USA | Parkinson disease | GROUP 1:60.5 (9.7) GROUP 2: 60 (10.3) | GROUP 1:63 (58.3)  GROUP 2: 74 (64.9) | GROUP 1:1.5 (1.5)  GROUP 2: 1.7 (1.6) | 104w | Pramipexole+open LD | Levodopa+open LD | incidence of dyskinesia;UPDRS III-motor;UPDRS-II(ADL);UPDRS-total;incidence of motor fluctuations | N/A |
|  |  |  |  |  |  |  |  | dose:1.5-4.5mg |  |  |  |
|  |  |  |  |  |  |  |  | freq: - |  |  |  |
|  |  |  |  |  |  |  |  | length of intervention: 288weeks |  |  |  |
| Noyes (Noyes et al., 2006) | 2006 | USA | Parkinson disease | GROUP 1:61.5 (10.1) GROUP 2: 60.9 (10.5) | GROUP 1:96 (63.6)  GROUP 2: 99 (66) | GROUP 1:1.5 (1.4)  GROUP 2: 1.8 (1.7) | 208w | Pramipexole | Levodopa | incidence of dyskinesia;incidence of motor fluctuations | Somnolence; Edema; Dyskinesias |
|  |  |  |  |  |  |  |  | dose:- |  |  |  |
|  |  |  |  |  |  |  |  | freq: - |  |  |  |
|  |  |  |  |  |  |  |  | length of intervention:192 weeks |  |  |  |
| Holloway (Holloway et al., 2004) | 2004 | USA | Parkinson disease | GROUP 1:61.1 (9.6) GROUP 2: 60.8 (9.8) | GROUP 1:50 (60.2)  GROUP 2: 68 (68) | GROUP 1:1.4 (1.3)  GROUP 2: 1.8 (1.7) | 208w | Pramipexole+open LD | Levodopa+open LD | incidence of dyskinesia;Levodopa equivalent doses ;UPDRS-II(ADL);UPDRS III-motor;UPDRS-total;incidence of motor fluctuations | Edema; Peripheral; edema; Somnolence; Hallucination; Cellulitis; Urinary frequency; Hernia |
|  |  |  |  |  |  |  |  | dose:0.5-1.5mg |  |  |  |
|  |  |  |  |  |  |  |  | freq:3 times per day |  |  |  |
|  |  |  |  |  |  |  |  | length of intervention:192 weeks |  |  |  |
| Holloway(Parkinson Study, 2000) | 2000 | USA | Parkinson disease | GROUP 1:61.5 (10.1)  GROUP 2: 50.9 (10.5) | GROUP 1:96 (63.6)  GROUP 2: 99 (66) | GROUP 1:1.5 (1.4)  GROUP 2: 1.8 (1.7) | 94w | Pramipexole;Pramipexole+openLD | Levodopa+open LD | incidence of dyskinesia;Levodopa equivalent doses ;UPDRS-II(ADL);UPDRS III-motor;UPDRS-total;incidence of motor fluctuations | Somnolence; Hallucination; Generalized edema; Peripheral edema; Nausea; Diziness; Insomnia; Headache; Constipation; Depression; Abnormal dreams; Anxiety; Postural hypotension |
|  |  |  |  |  |  |  |  | dose:0.25-1.5mg |  |  |  |
|  |  |  |  |  |  |  |  | freq:3 times per day |  |  |  |
|  |  |  |  |  |  |  |  | length of intervention:94 weeks |  |  |  |
| Rascol (Rascol et al., 2000) | 2000 | France | Parkinson disease | GROUP 1:63 (9) GROUP 2: 63 (9) | GROUP 1:113 (63.1)  GROUP 2: 52 (58.4) | GROUP 1:2.5 (2.8)  GROUP 2: 2.4 (2.3) | 260w | ropinirole ;Ropinirole+open LD | Levodopa+open LD | incidence of dyskinesia;Levodopa equivalent doses ;UPDRS III-motor;UPDRS-II(ADL);incidence of motor fluctuations | Nausea; Somnolence; Insomnia; Aggravated Parkinson's disease; Dyspepsia; Dizziness; Hallucinations; Vomiting; Tremor; Abdominal pain; Depression; Headache; Edema of the legs; Ataxia; Anxiety; Postural hypotension; Constipation; Dyskinesia;Dystonia; Increased sweating |
|  |  |  |  |  |  |  |  | dose:0.25-8mg |  |  |  |
|  |  |  |  |  |  |  |  | freq:3 times per day |  |  |  |
|  |  |  |  |  |  |  |  | length of intervention:260 weeks |  |  |  |
| Watts (Watts et al., 2010) | 2010 | USA | Parkinson disease | GROUP 1:61.4 (7.0) GROUP 2: 62.1 (7.2) | GROUP 1:60 (58)  GROUP 2: 74 (71) | GROUP 1:2.7 (2.1)  GROUP 2: 2.7 (2.4) | 2w | Ropinirole | Levodopa | incidence of dyskinesia;Levodopa equivalent doses ;UPDRS III-motor;UPDRS-II(ADL) | Nausea; Dizziness; Insomnia; Back Pain;Arthralgia; Somnolence; Fatigue; Pain in extremity |
|  |  |  |  |  |  |  |  | dose:2-24mg |  |  |  |
|  |  |  |  |  |  |  |  | freq: 1 times per day |  |  |  |
|  |  |  |  |  |  |  |  | length of intervention: 104weeks |  |  |  |
| Rascol (Rascol et al., 1998) | 1998 | France | Parkinson disease | GROUP 1:63 (9)  GROUP 2: 63 (9) | GROUP 1:113 (63.1)  GROUP 2: 51 (89) | GROUP 1:2.5 (2.8)  GROUP 2: 2.4 (2.3) | NA | Ropinirole | Levodopa | incidence of dyskinesia;Levodopa equivalent doses ;UPDRS III-motor | Nausea; Insomnia; Somnolence; Dizziness; Dyspepsia; Headache; Vomiting; Abdominal pain; Psychiatricsymptoms; Tremor; Anxiety; Anorexia; Postural hypotension; Increased; sweating; Abnormal involuntary movements;Depression |
|  |  |  |  |  |  |  |  | dose:0.25-8mg |  |  |  |
|  |  |  |  |  |  |  |  | freq: 3 times per day |  |  |  |
|  |  |  |  |  |  |  |  | length of intervention:24 weeks |  |  |  |
| Whone(Whone et al., 2003) | 2003 | UK | Parkinson disease | GROUP 1:61.0 (8.6) GROUP 2: 59.9 (9.23) | GROUP 1:56 (64.4)  GROUP 2: 53 (70.7) | GROUP 1:1.3 (0.57) GROUP2:1.36 (0.55) | 104w | Ropinirole | Levodopa | incidence of dyskinesia;Levodopa equivalent doses ;UPDRS III-motor | Nausea; Somnolence; Injury; Dizziness;Fatigue; Insomnia; Upper respiratory tract infection; Pain;Back; pain; Arthralgia; Headache; Edema; Dyspepsia; Tremor; Constipation; Abdominal; pain; Myalgia |
|  |  |  |  |  |  |  |  | dose:0.25-8mg |  |  |  |
|  |  |  |  |  |  |  |  | freq: 3 times per day |  |  |  |
|  |  |  |  |  |  |  |  | length of intervention:104 weeks |  |  |  |
| Hauser(Hauser et al., 2007) | 2007 | USA | Parkinson disease | GROUP 1:62.5 (8.0) GROUP 2: 61.5 (9.7) | GROUP 1:22 (52.4)  GROUP 2: 18 (66.7) | GROUP 1:2.1 (1.8)  GROUP 2: 2.24 (2.17) | 520w | Ropinirole | Levodopa | incidence of dyskinesia;Levodopa equivalent doses ;UPDRS III-motor;UPDRS-II(ADL);incidence of motor fluctuations | N/A |
|  |  |  |  |  |  |  |  | dose:- |  |  |  |
|  |  |  |  |  |  |  |  | freq:- |  |  |  |
|  |  |  |  |  |  |  |  | length of intervention:480 weeks |  |  |  |
| Adler (Adler et al., 1997) | 1997 | USA | Parkinson disease | GROUP 1:62 (10.57) GROUP 2: 63.8 (10.63) | GROUP 1:70 (60.3)  GROUP 2: 80 (64) | GROUP 1:2.05 (1.71) GROUP 2: 1.90 (1.61) | NA | Ropinirole | Placebo | UPDRS III-motor | Nausea; Dizziness; Somnolence; Headache; Upper respiratory tract; Confusion; Amnesia; Concentration; impairment; Depression; Hallucination; Delusion; Illusion infection; Insomnia; Constipation; Syncope |
|  |  |  |  |  |  |  |  | dose:0.25-8mg |  |  |  |
|  |  |  |  |  |  |  |  | freq: 3 times per day |  |  |  |
|  |  |  |  |  |  |  |  | length of intervention:24 weeks |  |  |  |
| Jankovic (Jankovic et al., 2007) | 2007 | USA | Parkinson disease | GROUP 1:62.0 (10.3) GROUP 2: 64.5 (10.7) | GROUP 1:123 (68)  GROUP 2: 58 (60) | GROUP 1:1.3 (1.3)  GROUP 2: 1.4 (1.3) | 4w | Transdermal Rotigotine | Placebo | UPDRS III-motor;UPDRS-II(ADL) | Application site disorders; Fatigue; Pain;Leg pain; Dizziness; Headaches; Tremor; Parkinsonism aggravated; Nausea; Vomiting; Constipation; Dyspepsia; Diarrhea; Arthralgia; Back pain; Skeletal pain; Somnolence Insomnia; Coughing; Upper respiratory tract infection; Sinusitis; Rash |
|  |  |  |  |  |  |  |  | dose:2-6mg |  |  |  |
|  |  |  |  |  |  |  |  | freq: 24h |  |  |  |
|  |  |  |  |  |  |  |  | length of intervention:27 weeks |  |  |  |
| Rascol(Rascol et al., 2006) | 2006 | France | Parkinson disease | GROUP 1：62.4 (9.5) GROUP 2: 62.3 (10.3) | GROUP 1:116 (58.9)  GROUP 2: 128 (62.7) | GROUP 1：2.0 (1.8) GROUP 2: 2.0 (2.0) | NA | Piribedil | Placebo | UPDRS III-motor;UPDRS-II(ADL) | Nausea; Hypertension; Dizziness; Anxiety; Hypotension postural; Insomnia; Constipation; Depression; Somnolence; Edema periphera; Abdominal pain |
|  |  |  |  |  |  |  |  | dose:50-300mg |  |  |  |
|  |  |  |  |  |  |  |  | freq: - |  |  |  |
|  |  |  |  |  |  |  |  | length of intervention:42 weeks |  |  |  |
| Marek(Parkinson Study, 2002) | 2002 | USA | Parkinson disease | GROUP 1:61.9 (10.8) GROUP 2: 60.1 (11.1) | GROUP 1:28 (66)  GROUP 2: 24 (59) | GROUP 1:1.3 (1.4)  GROUP 2: 1.6 (1.9) | 208w | Pramipexole | Levodopa | UPDRS III-motor;UPDRS-total | N/A |
|  |  |  |  |  |  |  |  | dose:0.5-1.5mg |  |  |  |
|  |  |  |  |  |  |  |  | freq:3 times per day |  |  |  |
|  |  |  |  |  |  |  |  | length of intervention:192 weeks |  |  |  |
| Wong(Wong et al., 2003) | 2003 | China | Parkinson disease | GROUP 1:58.84 (1.28) GROUP 2: 60.94 (1.11) | GROUP 1:48 (65.8)  GROUP 2: 56 (72.7) | GROUP 1:4.49 (0.4) GROUP 2: 4.33 (0.36) | NA | Pramipexole;Pramipexole+LD | Placebo;Placebo+ LD | UPDRS-II(ADL);UPDRS III-motor | Dizziness; Constipation; Nausea;Dry mouth; Dyskinesia; Hallucinations; Somnolence;Tremor |
|  |  |  |  |  |  |  |  | dose:0.125-1.5mg |  |  |  |
|  |  |  |  |  |  |  |  | freq: 3 times per day |  |  |  |
|  |  |  |  |  |  |  |  | length of intervention:15w |  |  |  |
| Kieburtz(Kieburtz et al., 1997) | 1997 | USA | Parkinson disease | GROUP 1:62.8 (11.4) GROUP 2: 60.4 (12.0) | GROUP 1:38 (69.1)  GROUP 2: 32 (62.7) | GROUP 1:2.2 (1.8)  GROUP 2: 1.7 (1.5) | 11w | Pramipexole | Placebo | UPDRS-total; | Somnolence; Dizziness; Nausea; Musculoskeletal pain; Headache; Constipation; Iasomnia;Fatigue; Hallucination; Confusion |
|  |  |  |  |  |  |  |  | dose:0.125-2.0mg |  |  |  |
|  |  |  |  |  |  |  |  | freq: 3 times per day |  |  |  |
|  |  |  |  |  |  |  |  | length of intervention:10w |  |  |  |
| Kieburtz(Kieburtz, 2011) | 2011 | USA | Parkinson disease | GROUP 1:64.1 (9.8) GROUP 2: 61.2 (11.0) | GROUP 1:57 (71.3)  GROUP 2: 58 (75.3) | GROUP 1:1.1 (1.3)  GROUP 2: 1.1 (1.5) | 1w | Pramipexole | Placebo | UPDRS-total; | Somnolence; Fatique; Dizziness; Headache; Insomnia; Abnormal Dreams;Nausea; Constipation;Peripheral Edema |
|  |  |  |  |  |  |  |  | dose:0.5mg;0.5mg;0.75mg; |  |  |  |
|  |  |  |  |  |  |  |  | freq: 2 times per day;3 times per day;2 times per day |  |  |  |
|  |  |  |  |  |  |  |  | length of intervention: 12 weeks |  |  |  |
| Zhang(Zhang et al., 2016) | 2016 | China | Parkinson disease | GROUP 1:59.7 (10.1) GROUP 2: 59.1 (10.3) | GROUP 1:76 (61.8)  GROUP 2: 74 (59.4) | GROUP 1:1.08 (1.27) GROUP 2: 0.94 (1.17) | 4w | Rotigotine | Placebo | impulse compulsive behaviors | Nausea; Dizziness; Pruritus; omnolence;Erythema; Vomiting |
|  |  |  |  |  |  |  |  | dose:2-8mg |  |  |  |
|  |  |  |  |  |  |  |  | freq: 24h |  |  |  |
|  |  |  |  |  |  |  |  | length of intervention:28 weeks |  |  |  |

# Supplementary Table 2. Tests for Publication Bias

**Incidence of dyskinesia**

**Egger's test**

| Std_Eff | Coef. | Std. Err. | t | P>\|t\| | [95% Conf. Interval] | |
| --- | --- | --- | --- | --- | --- | --- |
| slope | -1.595663 | .2711672 | -5.88 | 0.000 | -2.173642 | -1.017684 |
| bias | .9004501 | .6105465 | 1.47 | 0.161 | -.4008991 | 2.201799 |

**Incidence of motor fluctuations**

**Egger's test**

| Std_Eff | Coef. | Std. Err. | t | P>\|t\| | [95% Conf. Interval] | |
| --- | --- | --- | --- | --- | --- | --- |
| slope | -.729916 | .1593469 | -4.58 | 0.006 | -1.13953 | -.3203018 |
| bias | .4391504 | .5637686 | 0.78 | 0.471 | -1.010063 | 1.888364 |

**UPDRS-II(ADL)**

**Egger's test**

| Std_Eff | Coef. | Std. Err. | t | P>\|t\| | [95% Conf. Interval] | |
| --- | --- | --- | --- | --- | --- | --- |
| slope | -2.334388 | .8280094 | -2.82 | 0.014 | -4.123193 | -.5455822 |
| bias | 1.729164 | 1.335613 | 1.29 | 0.218 | -1.156252 | 4.614579 |

**UPDRS-motor**

**Egger's test**

| Std_Eff | Coef. | Std. Err. | t | P>\|t\| | [95% Conf. Interval] | |
| --- | --- | --- | --- | --- | --- | --- |
| slope | -4.855367 | 3.414387 | -1.42 | 0.174 | -12.09354 | 2.382809 |
| bias | 1.603233 | 2.340301 | 0.69 | 0.503 | -3.357983 | 6.564449 |

**UPDRS-total**

**Egger's test**

| Std_Eff | Coef. | Std. Err. | t | P>\|t\| | [95% Conf. Interval] | |
| --- | --- | --- | --- | --- | --- | --- |
| slope | -8.402739 | 3.156315 | -2.66 | 0.056 | -17.16607 | .3605969 |
| bias | 2.968996 | 2.013116 | 1.47 | 0.214 | -2.620309 | 8.558301 |

# Supplementary Figure 1. Risk of bias graph

**
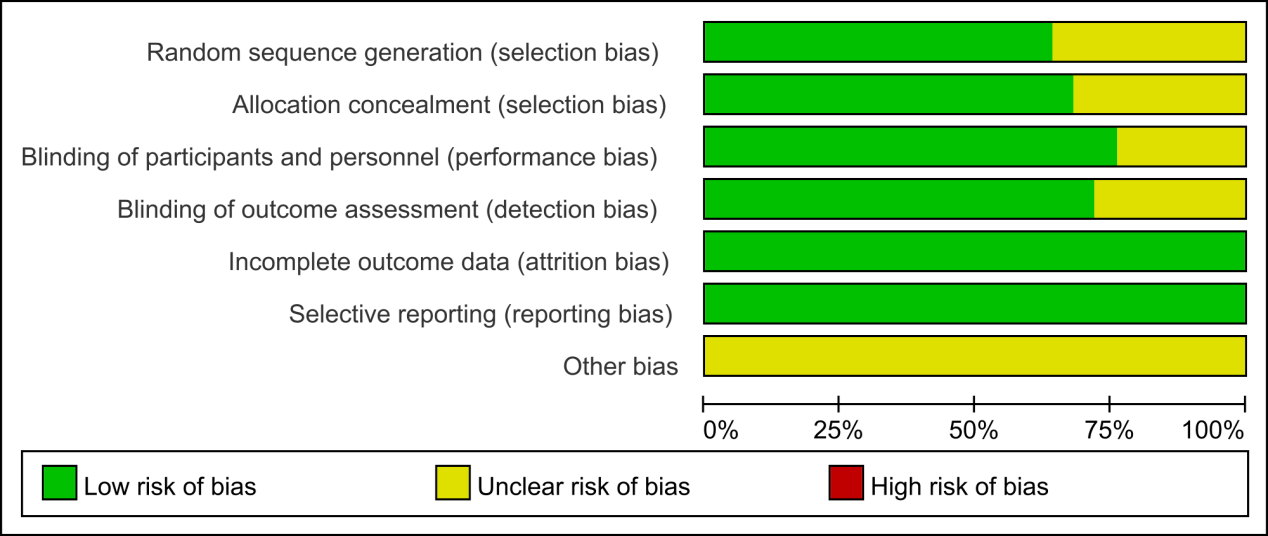
**Supplementary Figure 1. Risk of bias graph

# Supplementary Figure 2. Risk of bias summary


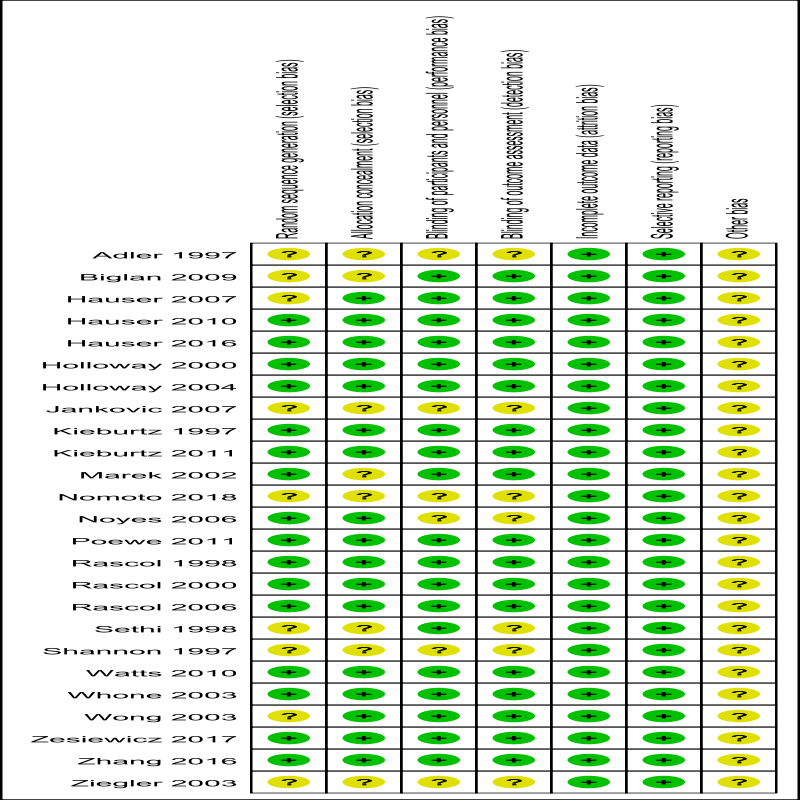


Supplementary Figure 2. Risk of bias summary

# Supplementary Figure 3 Sensitivity analysis

**
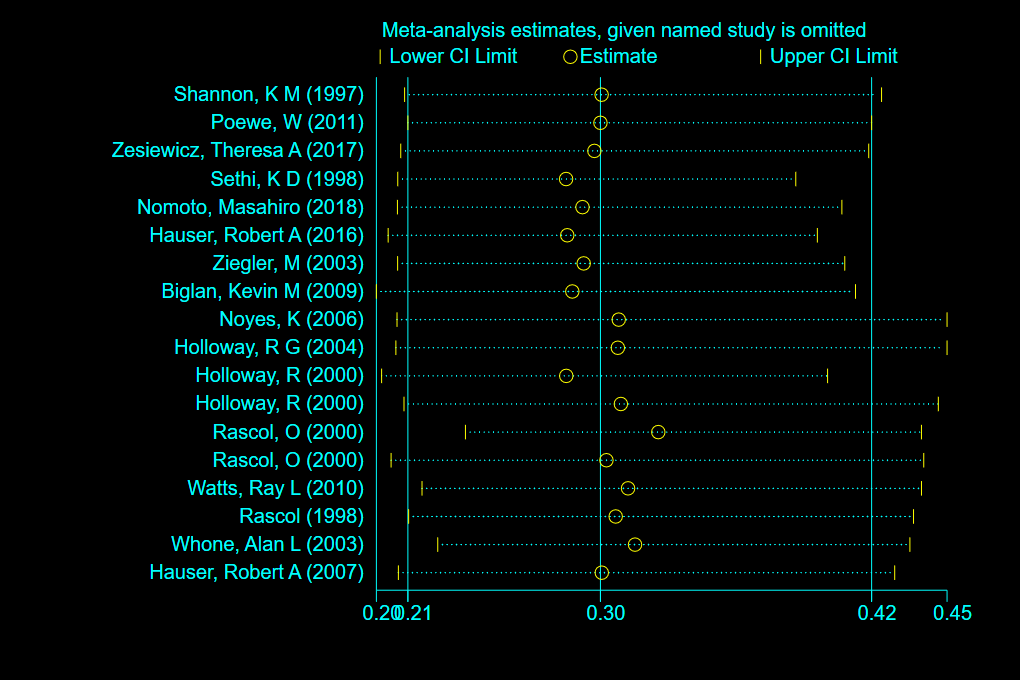
**Incidence of dyskinesia

**
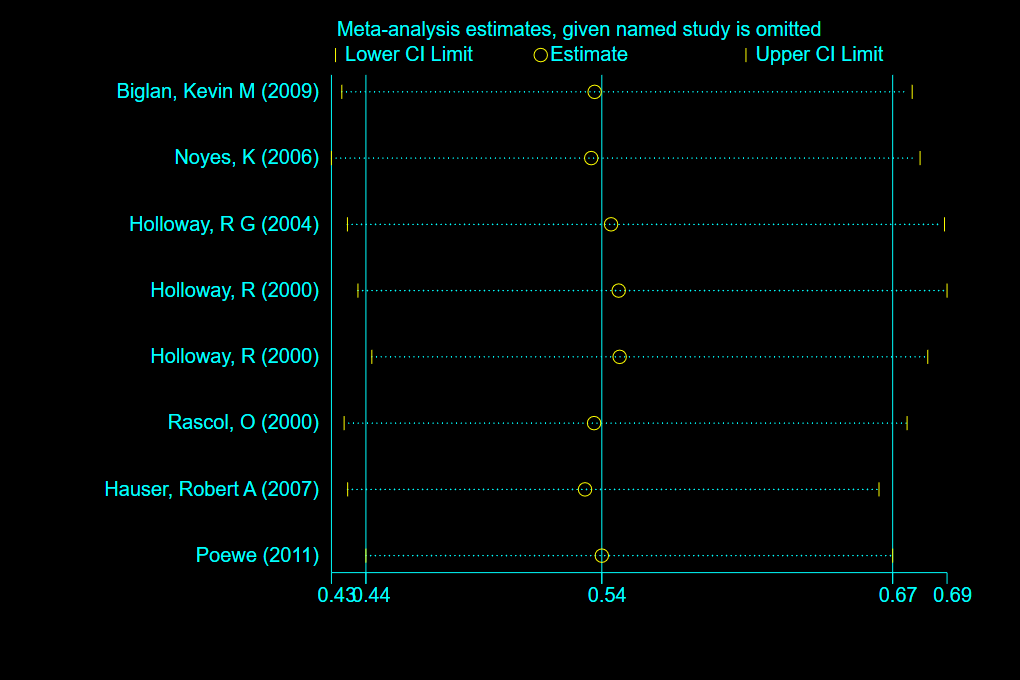
**Incidence of motor fluctuations

**
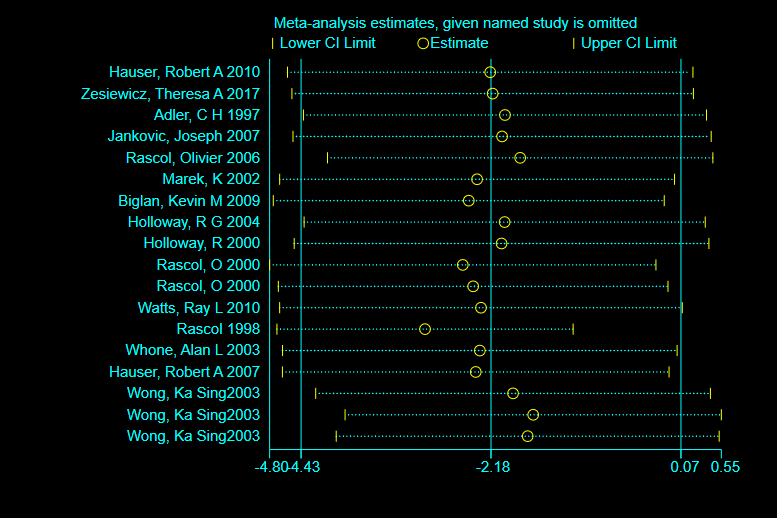
**UPDRS-motor

**
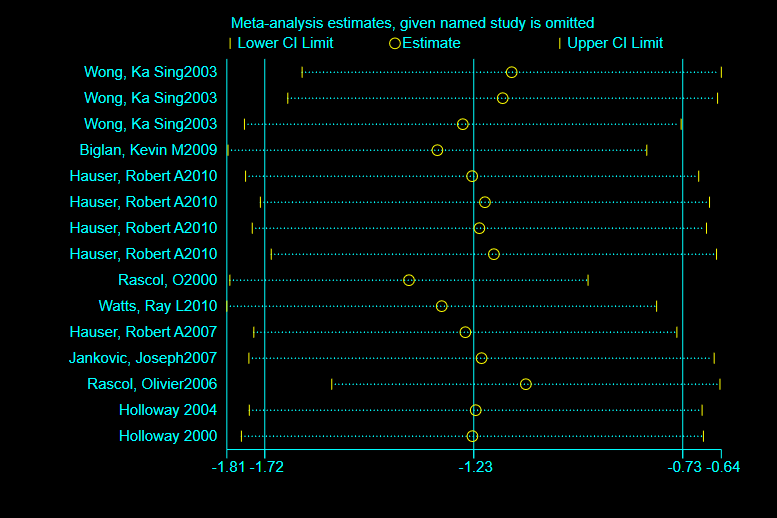
**UPDRS-II(ADL)

**
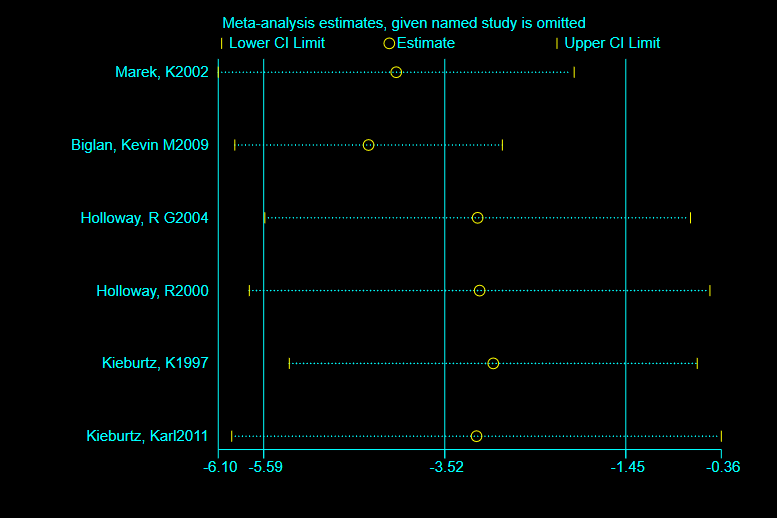
**UPDRS-total

Supplementary Figure 3. Sensitivity analysis of different outcomes

# Supplementary references

Adler, C.H., Sethi, K.D., Hauser, R.A., Da Vis, T.L., and Neurology, C.O.B.J. (1997). Ropinirole for the treatment of early Parkinson's disease. *Neurology* 49(2)**,** 393-399. doi: 10.1212/wnl.49.2.393.

Hauser, R.A., Rascol, O., Korczyn, A.D., Jon Stoessl, A., Watts, R.L., Poewe, W., et al. (2007). Ten-year follow-up of Parkinson's disease patients randomized to initial therapy with ropinirole or levodopa. *Mov Disord* 22(16)**,** 2409-2417. doi: 10.1002/mds.21743.

Hauser, R.A., Schapira, A., Rascol, O., Barone, P., and Poewe, W.J.M.D. (2010). Randomized, double-blind, multicenter evaluation of pramipexole extended release once daily in early Parkinson's disease. *Mov Disord* 25(15)**,** 2542-2549. doi: 10.1002/mds.23317.

Hauser, R.A., Slawek, J., Barone, P., Dohin, E., and Bauer, L.J.B.N. (2016). Evaluation of rotigotine transdermal patch for the treatment of apathy and motor symptoms in Parkinson's disease. *BMC Neurol* 16(1)**,** 90. doi: 10.1186/s12883-016-0610-7.

Holloway, R.G., Shoulson, I., Fahn, S., Kieburtz, K., Lang, A., Marek, K., et al. (2004). Pramipexole vs levodopa as initial treatment for Parkinson disease: a 4-year randomized controlled trial. *Arch Neurol* 61(7)**,** 1044-1053. doi: 10.1001/archneur.61.7.1044.

Jankovic, J., Watts, R.L., Martin, W., and Boroojerdi, B. (2007). Transdermal rotigotine: double-blind, placebo-controlled trial in Parkinson disease. *Arch Neurol* 64(5)**,** 676-682. doi: 10.1001/archneur.64.5.676.

Kieburtz, Karl, Shoulson, and Association, I.J.J.J.o.t.A.M. (1997). Safety and efficacy of pramipexole in early Parkinson disease. *Jama-Journal of the American Medical Association* 278(2)**,** 125-130.

Kieburtz, K. (2011). Twice-daily, low-dose pramipexole in early Parkinson's disease: a randomized, placebo-controlled trial. *Mov Disord* 26(1)**,** 37-44. doi: 10.1002/mds.23396.

Nomoto, M., Iwaki, H., Kondo, H., and Sakurai, M. (2018). Efficacy and safety of rotigotine in elderly patients with Parkinson's disease in comparison with the non-elderly: a post hoc analysis of randomized, double-blind, placebo-controlled trials. *J Neurol* 265(2)**,** 253-265. doi: 10.1007/s00415-017-8671-0.

Noyes, K., Dick, A.W., and Holloway, R.G. (2006). Pramipexole versus levodopa in patients with early Parkinson's disease: effect on generic and disease-specific quality of life. *Value Health* 9(1)**,** 28-38. doi: 10.1111/j.1524-4733.2006.00078.x.

Parkinson Study, G. (2000). Pramipexole vs levodopa as initial treatment for Parkinson disease: A randomized controlled trial. Parkinson Study Group. *Jama* 284(15)**,** 1931-1938. doi: 10.1001/jama.284.15.1931.

Parkinson Study, G. (2002). Dopamine transporter brain imaging to assess the effects of pramipexole vs levodopa on Parkinson disease progression. *Jama* 287(13)**,** 1653-1661. doi: 10.1001/jama.287.13.1653.

Parkinson Study Group, C.C.I. (2009). Long-term effect of initiating pramipexole vs levodopa in early Parkinson disease. *Arch Neurol* 66(5)**,** 563-570. doi: 10.1001/archneur.66.1.nct90001.

Poewe, W., Rascol, O., Barone, P., Hauser, R.A., Mizuno, Y., Haaksma, M., et al. (2011). Extended-release pramipexole in early Parkinson disease: a 33-week randomized controlled trial. *Neurology* 77(8)**,** 759. doi: 10.1212/WNL.0b013e31822affb0.

Rascol, O., Brooks, D.J., Brunt, E.R., Korczyn, A.D., Poewe, W.H., and Stocchi, F.J.M.D. (1998). Ropinirole in the treatment of early Parkinson's disease: A 6-month interim report of a 5-year levodopa-controlled study. *Mov Disord* 13(1)**,** 39-45. doi: 10.1002/mds.870130111.

Rascol, O., Brooks, D.J., Korczyn, A.D., De Deyn, P.P., Clarke, C.E., and Lang, A.E. (2000). A five-year study of the incidence of dyskinesia in patients with early Parkinson's disease who were treated with ropinirole or levodopa. *N Engl J Med* 342(20)**,** 1484-1491. doi: 10.1056/nejm200005183422004.

Rascol, O., Dubois, B., Caldas, A.C., Senn, S., Del Signore, S., and Lees, A. (2006). Early piribedil monotherapy of Parkinson's disease: A planned seven-month report of the REGAIN study. *Mov Disord* 21(12)**,** 2110-2115. doi: 10.1002/mds.21122.

Sethi, K.D.J.J.N. (1998). Ropinirole for the treatment of early Parkinson disease: a 12-month experience. Ropinirole Study Group. *Arch Neurol* 55(9)**,** 1211-1216. doi: 10.1001/archneur.55.9.1211.

Shannon, K.M., Bennett, J.P., Jr., and Friedman, J.H. (1997). Efficacy of pramipexole, a novel dopamine agonist, as monotherapy in mild to moderate Parkinson's disease. The Pramipexole Study Group. *Neurology* 49(3)**,** 724-728. doi: 10.1212/wnl.49.3.724.

Watts, R.L., Lyons, K.E., Pahwa, R., Sethi, K., Stern, M., Hauser, R.A., et al. (2010). Onset of dyskinesia with adjunct ropinirole prolonged-release or additional levodopa in early Parkinson's disease. *Mov Disord* 25(7)**,** 858-866. doi: 10.1002/mds.22890.

Whone, A.L., Watts, R.L., Stoessl, A.J., Davis, M., Reske, S., Nahmias, C., et al. (2003). Slower progression of Parkinson's disease with ropinirole versus levodopa: The REAL-PET study. *Ann Neurol* 54(1)**,** 93-101. doi: 10.1002/ana.10609.

Wong, K.S., Lu, C.S., Shan, D.E., Yang, C., and Mok, V.J.J.o.t.N.S. (2003). Efficacy, safety, and tolerability of pramipexole in untreated and levodopa-treated patients with Parkinson's disease. *J Neurol Sci* 216(1)**,** 81-87. doi: 10.1016/s0022-510x(03)00217-x.

Zesiewicz, T.A., Chriscoe, S., Jimenez, T., Upward, J., and VanMeter, S. (2017). A fixed-dose, dose-response study of ropinirole prolonged release in early stage Parkinson's disease. *Neurodegener Dis Manag* 7(1)**,** 49-59. doi: 10.2217/nmt-2016-0039.

Zhang, Z.X., Shang, H.F., Hu, X., Chen, S., Zhao, Z., Du, X., et al. (2016). Rotigotine transdermal patch in Chinese patients with early Parkinson's disease: A randomized, double-blind, placebo-controlled pivotal study. *Parkinsonism Relat Disord* 28**,** 49-55. doi: 10.1016/j.parkreldis.2016.04.022.

Ziegler, M., Castro-Caldas, A., Signore, S.D., and Rascol, O.J.M.D. (2003). Efficacy of piribedil as early combination to levodopa in patients with stable Parkinson's disease: a 6-month, randomized, placebo-controlled study. *Movement Disorders* 18(4)**,** 418–425. doi: 10.1002/mds.10359.
